# Comparative Evaluation of Real-Time PCR, Immunochromatographic Assay, and Modified Carbapenem Inactivation Method for Carbapenemase Detection in Enterobacterales Isolates

**DOI:** 10.3390/ijms27125454

**Published:** 2026-06-17

**Authors:** Elif Tuğçe Güner, Cemal Çiçek, Ayfer Bakır, Murat Aral

**Affiliations:** Department of Medical Microbiology, University of Health Science Ankara Etlik City Hospital, 06170 Ankara, Türkiye; drcemal06@gmail.com (C.Ç.); dr.ayfer.bakir@gmail.com (A.B.); aralmurat@hotmail.com (M.A.)

**Keywords:** carbapenemase, carbapenem-resistant Enterobacterales, carbapenem inactivation method, immunochromatographic assay, antimicrobial resistance, diagnostic algorithm, OXA-48

## Abstract

Carbapenem-resistant Enterobacterales represent a major global public health concern, and the rapid and reliable detection of carbapenemase production is of critical importance. In this study, real-time PCR was used as the reference method for carbapenemase detection, and the performance of immunochromatographic and phenotypic methods was comparatively evaluated. A total of 96 carbapenem-resistant Enterobacterales isolates were included, and all were identified as carbapenemase producers by the reference method. Diagnostic performance was assessed using positive percent agreement (PPA) and overall percent agreement (OPA); negative percent agreement and negative predictive value could not be calculated due to the absence of negative isolates. The immunochromatographic test showed complete agreement with real-time PCR in detecting carbapenemase production (PPA and OPA: 100%). At the gene level, sensitivity and specificity were 100% for OXA-48 and NDM, while sensitivity for KPC was 91.7%. In the modified carbapenem inactivation method (mCIM), PPA and OPA values were 97.9% due to false-negative results observed in two isolates producing OXA-48 and NDM by the reference method, and its performance was slightly lower than that of the immunochromatographic method. These findings indicate that the immunochromatographic method is a rapid, reliable, and practical option for carbapenemase detection, particularly in OXA-48-endemic intensive care settings where rapid identification is critical for timely infection control and appropriate antimicrobial therapy.

## 1. Introduction

The increasing prevalence of carbapenem resistance among bacteria belonging to the Enterobacterales family has become one of the most critical challenges directly affecting clinical microbiology practice today. The loss of efficacy of carbapenems, which have long been considered the last-line treatment for severe Gram-negative infections, has led to increased morbidity and mortality, particularly in hospital settings [1]. Among the mechanisms responsible for this resistance, carbapenemase production is of particular importance due to its high transmissibility and significant clinical impact [2,3]. Carbapenemase-producing Enterobacterales (CPE) complicate individual patient management and may lead to difficult-to-control outbreaks in healthcare settings [4]. Infections caused by these isolates are associated with limited therapeutic options and require aggressive infection control measures. In particular, CPE infections in intensive care unit (ICU) patients are associated with significantly higher mortality rates and prolonged hospital stays, and have been identified as independent risk factors for outbreak propagation in critical care environments [5,6]. Therefore, the early and accurate detection of CPE has become a critical necessity from both clinical and epidemiological perspectives [3,7].

According to the Ambler classification, carbapenemase enzymes are mainly categorized into three groups: class A carbapenemases (*Klebsiella pneumoniae* carbapenemases [KPC]), class B metallo-β-lactamases (New Delhi metallo-β-lactamase [NDM], Verona integron-encoded metallo-β-lactamase [VIM], and IMP-type metallo-β-lactamase [IMP]), and class D carbapenemases (OXA-48-like class D β-lactamases). Although class C β-lactamases (AmpC enzymes) are also part of the Ambler classification, they do not possess clinically significant carbapenem-hydrolyzing activity in Enterobacterales and are therefore not considered true carbapenemases in this context. These enzymes hydrolyze carbapenems, leading to high-level β-lactam resistance in Enterobacterales species. Among them, NDM, KPC, and OXA-48-like enzymes are the most frequently encountered in clinical practice and pose the greatest diagnostic challenges [7,8]. Epidemiological studies conducted in Europe and the Mediterranean region have demonstrated that OXA-48-like carbapenemases are predominant, particularly in Türkiye. However, the increasing reports of NDM and KPC genes occurring either alone or in co-production with OXA-48 have added further complexity to diagnostic approaches. Isolates harboring multiple carbapenemase genes present additional challenges in both phenotypic and genotypic evaluations [9,10,11]. At the national and international level, leading clinical microbiology societies and guidelines recommend carbapenemase confirmatory testing for all carbapenem-resistant Enterobacterales isolates, with a preference for molecular or immunochromatographic methods given their superior sensitivity for OXA-48-like enzymes [7]. One of the main reasons why OXA-48-like carbapenemases are particularly problematic from a diagnostic perspective is their typically low-level carbapenem hydrolysis. This characteristic may result in false-negative results in routine susceptibility testing. Moreover, the interpretation of phenotypic tests becomes more complicated in co-producing isolates [12,13].

The rapid and reliable detection of carbapenemase production is essential for initiating appropriate antimicrobial therapy, implementing patient isolation, and ensuring timely infection control measures. Although molecular methods are considered the gold standard due to their ability to directly detect carbapenemase genes, their routine use may be limited by cost, technical infrastructure, and accessibility [14]. Therefore, immunochromatographic rapid diagnostic tests and phenotypic methods provide practical and accessible alternatives to molecular approaches. Their rapid turnaround time and ease of use make them valuable tools in clinical decision-making processes [15]. However, considerable variability exists among these methods in terms of performance and limitations, necessitating comparative evaluations [16].

The aim of this study was to comparatively evaluate the performance of immunochromatographic and phenotypic diagnostic methods, using real-time polymerase chain reaction (real-time PCR) as the reference method, in carbapenem-resistant Enterobacterales isolates, particularly those harboring single and multiple carbapenemase gene profiles, including OXA-48, NDM, and KPC.

## 2. Results

### 2.1. General Characteristics of the Isolates

In the analysis performed using the real-time PCR-based BD MAX™ Check-Points Carbapenemase Panel (BD MAX™ CPO) as the reference method, at least one carbapenemase gene was detected in all isolates included in the study, and all samples were classified as CPE-positive. Among the detected genes, OXA-48-like, NDM, and KPC were identified either as single genes or in multiple combinations.

**Figure 1:**
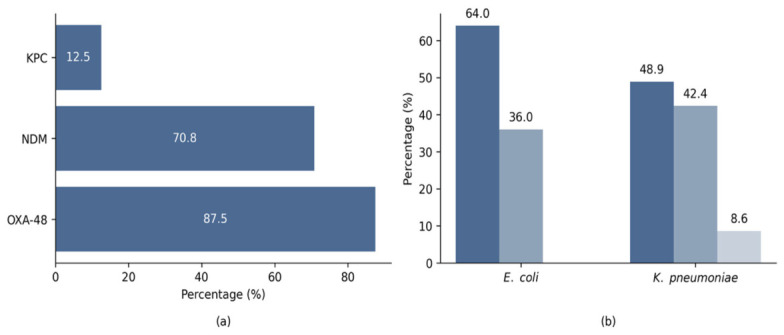
(**a**) Prevalence of OXA-48, NDM, and KPC carbapenemase genes among the tested isolates; (**b**) species-specific distribution of OXA-48, NDM, and KPC carbapenemase genes. Values are expressed as percentages. In panel (**b**), the dark blue, medium blue, and light gray bars represent OXA-48, NDM, and KPC, respectively.

OXA-48-like genes were detected in 87.5% (n = 84), NDM in 70.8% (n = 68), and KPC in 12.5% (n = 12) of the tested isolates (Figure 1a). When evaluated based on bacterial species, OXA-48 (64.0%) and NDM (36.0%) genes were identified in *Escherichia coli* isolates, whereas OXA-48 (48.9%) and NDM (42.4%) were predominant in *Klebsiella pneumoniae* isolates, with KPC detected at a lower frequency (8.6%) (Figure 1b).

VIM or IMP carbapenemases were not detected in any of the isolates by either the real-time PCR reference method or the immunochromatographic method using the RESIST-5 O.K.N.V.I. card test. A single carbapenemase gene was identified in 30.2% of the isolates, whereas multiple carbapenemase genes were detected in 69.8%. Co-production most frequently involved the combination of OXA-48 and NDM.

The general characteristics of the isolates included in the study are summarized in Table 1, where isolates are categorized according to single and multiple carbapenemase gene profiles. Percentages were calculated based on the total number of isolates (n = 96).

**Figure 2:**
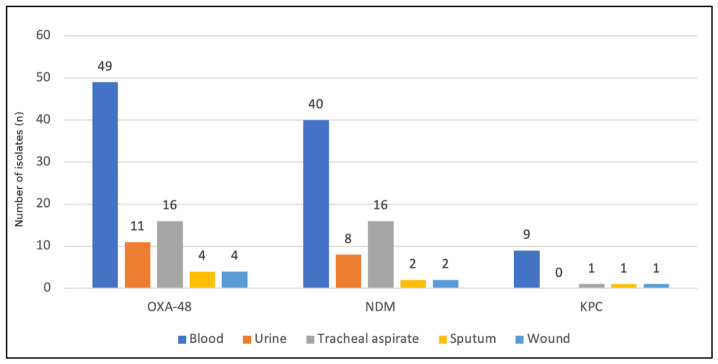
Distribution of clinical sample types among isolates harboring OXA-48, NDM and KPC carbapenemase genes. All values are presented as absolute isolate counts (n).

When the distribution of specimen types according to carbapenemase genes was examined, blood was found to be the most frequently isolated specimen type across all gene categories. In OXA-48- and NDM-positive isolates, blood samples were followed by tracheal aspirates and urine samples, while sputum and wound samples were detected at lower frequencies. KPC-producing isolates were limited in number and were predominantly identified from blood samples (Figure 2).

### 2.2. Agreement Between Real-Time PCR and the Immunochromatographic Assay

When the results of the real-time PCR reference method were compared with those of the RESIST-5 O.K.N.V.I. card test, the card test accurately detected the presence of carbapenemase in all isolates after an analysis time of approximately 15 min. Accordingly, the positive percent agreement (PPA) and overall percent agreement (OPA) values for the detection of carbapenemase presence were calculated as 100%. However, in the gene-level analysis, one of the multiple carbapenemase genes detected by the reference method in a single isolate could not be identified by the card test. Since no carbapenemase-negative isolates were identified by the reference method, negative percent agreement (NPA) and negative predictive value (NPV) could not be calculated.

### 2.3. Agreement Between Real-Time PCR and the Phenotypic Method

In the evaluation performed using the carbapenem inactivation method (mCIM), 94 of the 96 isolates were found to be positive, while 2 were negative. Accordingly, the PPA and OPA values of the mCIM test were calculated as 97.9%. In the two isolates that were false negative by the mCIM test, the reference method identified one as an NDM-producing *Escherichia coli* and the other as an OXA-48-producing *Klebsiella pneumoniae*, and both isolates were obtained from blood cultures. Both isolates were obtained from blood cultures. The meropenem MIC values for these two isolates, as determined by the BD Phoenix™ automated system, were 2 mg/L for the NDM-producing *Escherichia coli* and 4 mg/L for the OXA-48-producing *Klebsiella pneumoniae*, both representing low-level carbapenem resistance consistent with the known reduced hydrolytic activity of these enzymes, which likely contributed to the false-negative mCIM results. Since no carbapenemase-negative isolates were identified by the reference method, NPA and NPV could not be calculated.

### 2.4. Gene-Level Evaluation

In the genotypic analysis, partial genotypic discordance between the reference method and the card test was detected in one isolate. In this isolate, OXA-48 + NDM + KPC genes were identified by the reference method, whereas OXA-48 and NDM were detected by the card test, and the KPC gene was not identified. This isolate was not classified as false negative in terms of carbapenemase presence and was categorized as partial discordance at the gene level. For KPC detection, the sensitivity of the rapid test was calculated as 91.7%, while the specificity was 100%. No VIM or IMP carbapenemase positivity was detected in any of the isolates by either the real-time PCR-based BD MAX™ CPO reference method or the RESIST-5 O.K.N.V.I. card test. According to the BD MAX™ CPO reference method, the diagnostic performance of the RESIST-5 O.K.N.V.I. test according to carbapenemase type is summarized in Table 2.

The detailed carbapenemase gene profiles and comparative results of BD MAX™ CPO, RESIST-5, and mCIM for all 96 Enterobacterales isolates are provided in Table S1.

## 3. Discussion

The predominance of OXA-48 carbapenemases observed in this study is consistent with epidemiological patterns reported in Türkiye and the Eastern Mediterranean region and is supported by multicenter national cohort studies, genomic analyses, and global surveillance data [11,17,18,19]. The relatively high rate of NDM is also in line with recent studies from Türkiye reporting an increasing prevalence of NDM-producing strains, either in co-production with OXA-48 or independently [20]. The lower frequency of KPC-producing isolates identified in this study is consistent with the historically lower prevalence of KPC reported in Türkiye compared to Southern Europe and North America [21]. In the present study, single carbapenemase gene carriage in *E. coli* and *K. pneumoniae* isolates has largely been replaced by the presence of multiple gene combinations. The frequent co-detection of OXA-48 and NDM genes, in particular, suggests a shift in the molecular epidemiology of CPE isolates. This finding is in agreement with recent literature indicating a global increase in the co-occurrence of resistance genes [22,23,24]. The observed increase in multiple gene combinations may be associated with selective pressure driven by intensive antibiotic use in hospital settings, as well as epidemiological dynamics that facilitate the dissemination of resistant isolates within healthcare institutions, particularly considering that the isolates in this study were obtained from hospitalized patients [25].

When our findings were evaluated on a species basis, the more pronounced co-occurrence of multiple carbapenemase genes in *K. pneumoniae* suggests that this species may serve as an important reservoir for resistance genes. This observation may be related to the interaction of different bacterial species with plasmids carrying carbapenemase genes and to variability in their capacity to disseminate these plasmids [26,27]. The greater genomic plasticity of *K. pneumoniae* may confer a selective advantage for the accumulation of co-resistance traits under ongoing antimicrobial pressure [28,29,30]. These findings highlight the importance of species-based analyses in the epidemiological assessment of carbapenem resistance. Therefore, the rapid and reliable detection of carbapenemase production is of considerable importance for both monitoring the spread of resistance and ensuring the timely implementation of appropriate treatment strategies.

In this study, the complete agreement of the RESIST-5 O.K.N.V.I. lateral flow test with the real-time PCR method (PPA and OPA: 100%), along with its ability to accurately detect carbapenemase production in all isolates within approximately 15 min, indicates that this method may be used as a reliable screening tool not only in clinical microbiology laboratories but also in point-of-care settings, where clinicians could obtain results during the patient examination process. Previous studies have reported that RESIST-5 O.K.N.V.I. and similar lateral flow-based assays can detect clinically relevant carbapenemases, such as OXA-48, KPC, and NDM, with high accuracy [31,32,33,34]. According to our findings, the immunochromatographic method showed complete agreement with the reference method in detecting carbapenemase presence; however, partial discordance at the gene level was observed due to the inability to detect KPC in a single isolate producing multiple carbapenemases. While 100% sensitivity and specificity were achieved for OXA-48 and NDM, the sensitivity for KPC was 91.7%, with the only false-negative result identified in a triple co-producer isolate (OXA-48 + NDM + KPC). This isolate was not classified as false negative in terms of carbapenemase production, as two of the three genes were correctly identified. The failure to detect the KPC signal likely reflects a limitation in detailed genotypic differentiation rather than a misclassification of carbapenemase presence, as the other carbapenemase enzymes in the isolate were successfully identified and this would not directly affect clinical treatment decisions or infection control measures. Therefore, particularly in settings where molecular methods are limited by cost, infrastructure, or turnaround time, these rapid and easy-to-use immunochromatographic assays may serve not only as valuable laboratory screening tools but also as potential point-of-care diagnostic aids, facilitating earlier clinical decision-making and patient management. Furthermore, it has been previously reported that immunochromatographic assays may not detect all gene combinations simultaneously in isolates harboring multiple carbapenemase genes [35,36,37]. In particular, in cases of co-production involving NDM and OXA-48, some enzymes with low hydrolytic activity may be overlooked, and competitive interactions between different enzymes may play a role. A plausible mechanism is competitive epitope binding: in isolates co-expressing multiple carbapenemases, the immunochromatographic test strip may be saturated by the more abundantly expressed OXA-48 and NDM proteins, reducing the available binding capacity for KPC-specific antibodies. Additionally, differential gene expression levels among co-harbored resistance genes can result in KPC protein being present at sub-detectable concentrations, further contributing to reduced signal intensity [17,38]. On the other hand, studies evaluating isolates with multiple carbapenemase genes have also reported sensitivity and specificity rates approaching 100% for immunochromatographic methods [39,40,41,42]. The findings of our study are largely consistent with these data, except for the partial genotypic discordance observed in a single isolate. From a clinical and antimicrobial stewardship perspective, the 15 min turnaround time of the RESIST-5 O.K.N.V.I. test provides a significant advantage compared to the approximately 2.5 h required for BD MAX™ CPO and the overnight incubation required for the mCIM test. The rapid detection of CPE is crucial for preventing potential transmission associated with diagnostic delays [43]. Therefore, particularly in settings where molecular methods are limited by cost and time, we believe that these rapid and easy-to-use assays may contribute to treatment management in both laboratory and point-of-care settings. Based on our findings, we propose that in OXA-48-endemic ICU settings, immunochromatographic testing should be considered as the first-line confirmatory screening tool, effectively replacing CIM as the primary phenotypic method. A practical stepwise algorithm would involve: (1) immunochromatographic testing as first-line screening upon detection of carbapenem resistance; (2) CIM as an alternative only where immunochromatographic tests are unavailable; and (3) real-time PCR confirmation in cases of clinical discordance or when precise genotypic characterization is required for epidemiological purposes.

In this study, mCIM, evaluated as a phenotypic method, generally demonstrated high performance in detecting carbapenemase presence and showed consistency with the high PPA and OPA rates reported in the literature [44,45]. These findings support the use of mCIM as a practical and reliable phenotypic tool for carbapenemase screening. However, in our study, false-negative results were obtained with mCIM in two isolates in which real-time PCR detected OXA-48 production in one and NDM production in the other, indicating the limitations of this method. Previous studies have reported that the performance of mCIM may vary depending on the class of the carbapenemase enzyme [44,45]. It has been shown that OXA-48 and OXA-48-like enzymes may lead to higher rates of false-negative results in mCIM compared to other carbapenemases such as KPC or NDM [46,47,48]. In earlier studies, a substantial proportion of isolates yielding false-negative results in mCIM were found to harbor OXA-48 [49,50,51]. This discordance may be associated with the lower hydrolytic activity of these enzymes against carbapenems and their weak expression in certain isolates. Additionally, the presence of low minimum inhibitory concentration (MIC) values for carbapenems in some OXA-48-producing isolates may further complicate the detection of carbapenemase activity by phenotypic methods [17,51]. Consistent with our findings, false-negative results have also been reported in studies demonstrating high sensitivity and specificity of mCIM, particularly in the detection of metallo-β-lactamases such as NDM [50,52]. These false-negative results in mCIM may be attributed to heterogeneous expression levels, insufficient expression of hydrolytic activity under test conditions, and variable enzyme activity of NDM-producing isolates due to their dependence on metal ions [53].

According to the findings of this study, particularly in regions where OXA-48 is endemic and the prevalence of metallo-β-lactamases is high, when mCIM is used alone, negative results may be more appropriately interpreted in conjunction with immunochromatographic or molecular methods in the presence of strong clinical suspicion of carbapenemase production. Nevertheless, although the mCIM test is time-consuming due to its prolonged incubation period, it remains a useful method in laboratories processing a limited number of samples and where molecular tests are not routinely available, given its cost-effectiveness and ease of application. Specifically, mCIM may be most appropriate in resource-limited laboratory settings that lack access to molecular or immunochromatographic platforms, in low-throughput laboratories performing confirmatory testing on a small number of carbapenem-resistant isolates per day, and in settings where cost constraints preclude the use of commercial rapid tests. In such contexts, mCIM can serve as a simple, low-cost phenotypic confirmatory tool, provided that its limitations regarding OXA-48 and NDM detection are taken into account and results are interpreted with appropriate clinical suspicion.

This study has several limitations. The absence of carbapenemase-negative isolates in the reference method precluded the calculation of NPA and NPV. In addition, the limited number of isolates restricted the evaluation of rare gene combinations. Due to the presence of only twelve KPC-producing isolates, the statistical power for estimating sensitivity was limited. However, the wide CIs for KPC sensitivity reflects not an analytical shortcoming but a limitation inherent to the study design, consistent with the epidemiology of KPC in the region from which the isolates were obtained. Another important limitation is the restricted species diversity of the study population. Only two Enterobacterales species were included (*Klebsiella pneumoniae* and *Escherichia coli*), which limits the generalizability of the findings to other clinically relevant Enterobacterales species such as *Enterobacter cloacae*, *Klebsiella oxytoca*, *Serratia marcescens*, or *Citrobacter* spp. The diagnostic performance of the evaluated methods may differ in these species due to variations in outer membrane protein expression, efflux pump activity, and other non-carbapenemase resistance mechanisms that could influence phenotypic test results. Future studies should include a broader range of Enterobacterales species to validate and extend the current findings. To transparently reflect the statistical uncertainty arising from the small sample size, the results were reported together with Clopper–Pearson binomial CIs. A key design feature of this study is the inclusion of carbapenemase-producing isolates confirmed by the reference method. This approach was deliberately chosen to reflect the real-world clinical workflow in which confirmatory tests are applied to isolates identified as carbapenem-resistant by automated antimicrobial susceptibility systems. In this context, the primary research question of our study is not whether these tests can accurately identify carbapenemase-negative isolates, but rather which method provides the most accurate and rapid results among carbapenemase-producing isolates. Nevertheless, we acknowledge that prospective studies with a broader phenotypic spectrum are needed to fully characterize false-positive rates. Furthermore, the inclusion of isolates exclusively from patients hospitalized in the intensive care unit of a single tertiary-care center limits the generalizability of the findings to community-acquired infections or other healthcare settings; therefore, the results should primarily be interpreted in the context of similar hospital populations. Despite these limitations, the direct comparison of molecular, immunochromatographic, and phenotypic methods on the same isolates, the inclusion of isolates producing multiple carbapenemases enabling detailed gene-level evaluation, the application of binomial CIs and the transparent reporting of the kappa paradox represent key strengths of the study.

## 4. Materials and Methods

### 4.1. Study Design and Sample Collection

This study was designed as a single-center, prospective, analytical, and comparative laboratory study and was conducted at the Medical Microbiology Clinic of Ankara Etlik City Hospital. To compare the performance of different diagnostic methods for detecting carbapenemase production in *Enterobacterales* isolates, the molecular diagnostic method real-time PCR was accepted as the reference method, and the results of immunochromatographic and phenotypic methods were comparatively evaluated against this reference. These methods have mostly been evaluated separately in the literature in isolates carrying single genes or limited gene combinations [32,50,54]. In contrast, the present study aims to contribute to the literature by simultaneously comparing three different methods on the same isolates in an intensive care population in Türkiye, where OXA-48 predominance and high rates of multiple gene co-occurrence are observed, and by providing a detailed assessment of inter-method agreement and limitations, particularly in isolates harboring multiple carbapenemase genes.

A total of 96 Enterobacterales isolates (*Klebsiella pneumoniae*, n = 80; *Escherichia coli*, n = 16) were included in the study. These isolates were randomly selected from Enterobacterales isolates that had been isolated from various clinical specimens submitted from patients hospitalized in the intensive care unit to the Microbiology Laboratory of Ankara Etlik City Hospital between 1 January 2024 and 31 May 2024, and stored as part of the routine laboratory workflow. All included isolates were identified as carbapenem-resistant based on their antibiogram results. No prior power analysis was performed for sample size determination; the study sample was formed by randomly selecting eligible isolates received during the predefined study period. Only Enterobacterales isolates obtained from clinical specimens of adult patients (≥18 years) hospitalized in the intensive care unit were included. For duplicate isolates obtained from the same patient, only the first isolate chronologically was included in the study. The distinction between colonization and infection was not systematically assessed based on clinical data, and all isolates were evaluated as carbapenem-resistant Enterobacterales isolated from clinical specimens.

### 4.2. Bacterial Identification and Carbapenemase Detection Methods

Bacterial identification at the species level was performed using matrix-assisted laser desorption/ionization time-of-flight mass spectrometry (MALDI-TOF MS; Bruker MALDI Biotyper^®^ Sirius, Bruker Daltonics, Bremen, Germany). Antimicrobial susceptibility testing was carried out using the BD Phoenix™ NMIC-500 panel and the BD Phoenix™ M50 automated system (BD Diagnostic Systems, Sparks, MD, USA), in accordance with the recommendations and breakpoint criteria of the European Committee on Antimicrobial Susceptibility Testing (EUCAST). Using this method, in which MICs were determined, all isolates were identified as resistant to meropenem, imipenem, and ertapenem. Antimicrobial susceptibility testing was performed according to EUCAST recommendations, as they represent the routine standard for susceptibility testing and result interpretation in our laboratory and are the nationally adopted reference criteria in Türkiye. The isolates were stored at −80 °C in tryptic soy broth until real-time PCR, immunochromatographic, and phenotypic tests for the detection of carbapenemase production were performed. Prior to analysis, isolates were subcultured to obtain pure cultures, and all tests were conducted using fresh cultures. *Escherichia coli* ATCC 25922 was used as the quality control strain for all assays. In addition, positive controls for OXA-48, NDM, and KPC carbapenemases were included in each run of both the BD MAX™ CPO real-time PCR (BD Diagnostic Systems, Sparks, MD, USA), and the The RESIST-5 O.K.N.V.I. immunochromatographic assay (CORIS BioConcept, Gembloux, Belgium), using previously characterized reference strains, to ensure the validity and reproducibility of results across all testing sessions.

#### 4.2.1. Reference Method (Real-Time PCR)

Real-time PCR was used as the reference method for the detection of carbapenemase genes. Molecular analyses were performed on the BD MAX™ system using the BD MAX™ Check-Points CPO Assay kit (Check-Points, Wageningen, The Netherlands) provided by the manufacturer. This closed and fully automated system integrates nucleic acid extraction, amplification, and real-time detection steps on a single platform. The system is capable of simultaneously detecting *bla*_KPC, *bla*_NDM, *bla*_VIM, *bla*_IMP, and *bla*_OXA-48-like carbapenemase genes within approximately 2.5 h. According to the manufacturer’s data, the BD MAX™ CPO test demonstrates high diagnostic performance for the most commonly encountered carbapenemase types in clinical practice, namely KPC, NDM, and OXA-48-like enzymes, with reported sensitivity ranging from 88.5% to 100% and specificity from 98.6% to 100%. All tests were performed in accordance with the manufacturer’s instructions, and results were automatically analyzed using the system’s integrated software. The detection of at least one carbapenemase gene was considered sufficient for classifying an isolate as CPE [55].

#### 4.2.2. Immunochromatographic Assay

**Figure 3:**
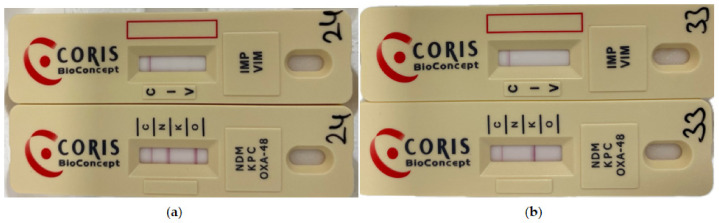
Detection of carbapenemase genes using the RESIST-5 O.K.N.V.I. immunochromatographic card test. (**a**) In the left cassette pair, positive bands for NDM and OXA-48 are observed in the lower cassette, (**b**) while in the right cassette pair, a positive band for KPC is observed in the lower cassette; no positivity is detected for IMP and VIM in the upper cassettes of both tests. C: control band; N: NDM; K: KPC; O: OXA-48; I: IMP; V: VIM.

The RESIST-5 O.K.N.V.I. card test, a lateral flow-based immunochromatographic method, was used for the detection of carbapenemase production. This test is a rapid, ready-to-use system capable of simultaneously detecting OXA-48, KPC, NDM, VIM, and IMP carbapenemases from bacterial colony samples through monoclonal antibodies labeled with colloidal gold nanoparticles. The test consists of two separate lateral flow cassettes, one targeting OXA-48, KPC, and NDM, and the other targeting VIM and IMP carbapenemases. All tests were performed using colony material obtained from fresh cultures in accordance with the manufacturer’s instructions, and results were interpreted within approximately 15 min. Positive results were determined by the presence of a colored band at the test line corresponding to the respective carbapenemase type (Figure 3). All results were independently evaluated simultaneously by two microbiology specialists. According to the manufacturer’s data, the test demonstrates sensitivity and specificity values of up to 100% for OXA-48, KPC, NDM, and IMP, whereas for VIM, sensitivity is reported as 93.5% and specificity as 100%.

#### 4.2.3. Phenotypic Method (Modified Carbapenem Inactivation Method [mCIM])

The modified carbapenem inactivation method (mCIM) was applied to evaluate phenotypic carbapenemase activity. The assay was performed and interpreted according to Clinical and Laboratory Standards Institute (CLSI) recommendations, which provide the standardized protocol for this phenotypic carbapenemase detection method. This method is based on the ability of the tested bacterium to inactivate a carbapenem antibiotic [54]. Test results were interpreted as positive or negative based on the presence and diameter of the inhibition zone. In this study, a 10 µg meropenem disk was used, and the test was considered positive when the inhibition zone diameter was less than 19 mm and/or when bacterial growth was observed within the inhibition zone. The standard CIM protocol was followed as described by van der Zwaluw et al. [56], with bacterial suspensions prepared at a 0.5 McFarland turbidity standard, incubated in 400 µL of sterile water at 35 °C for 2 h prior to disk placement, followed by overnight incubation of the test plate. mCIM results were evaluated only in terms of the presence of carbapenemase, and no genotypic differentiation was performed. All procedures and interpretations were carried out in accordance with the CLSI M100 guidelines [57]. EUCAST breakpoints were used exclusively for the initial antimicrobial susceptibility testing to define carbapenem resistance, as this is the guideline adopted by our institution for routine susceptibility reporting; CLSI guidelines were applied for the phenotypic CIM interpretation, consistent with the original CIM methodology described in the referenced literature [57,58].

This study was conducted in accordance with the Declaration of Helsinki and approved by the Scientific Research Evaluation and Ethics Committee of Ankara Etlik City Hospital (Approval No. AEŞH-BADEK-2024-982, 30 October 2024). The study was retrospective in design and used bacterial isolates that had been routinely collected and archived during standard laboratory workflow between January and May 2024. Study-specific analyses were performed after ethics committee approval had been obtained. Following Turkish legislation (Regulation on Clinical Trials of Medicinal Products for Human Use, Official Gazette No. 32203, 27 May 2023, Article 2/2), informed consent was waived because the study involved only anonymized archived isolates and did not require any additional patient intervention or sample collection.

### 4.3. Statistical Analysis

The results of the immunochromatographic assay and mCIM were compared with those of the BD MAX™ CPO panel. The diagnostic performance of the methods was evaluated by calculating PPA and OPA. Since no negative isolates were identified by the reference method, NPA and NPV could not be calculated. Inter-method agreement was analyzed using Cohen’s kappa coefficient. For the agreement between the reference method and the other methods, Cohen’s kappa coefficient was 0.00 (95% CI: approximately −0.10 to 0.10), indicating that the estimate was unstable due to the high prevalence of positive results (“kappa paradox”). Therefore, considering the limited ability of the kappa coefficient to reflect true agreement, PPA and OPA were used to describe the agreement between the methods [59]. For carbapenemase genes, sensitivity, specificity, PPV, NPV, and accuracy were calculated together with 95% CI. CIs were estimated using the Clopper–Pearson exact method based on the binomial distribution.

## 5. Conclusions

In conclusion, this study demonstrates that immunochromatographic tests represent a practical and reliable option for the detection of carbapenemase production, with a short turnaround time, and provide higher diagnostic performance compared to phenotypic CIM-based methods. Based on our findings, we propose a stepwise diagnostic algorithm for OXA-48-endemic ICU settings: (1) immunochromatographic testing as first-line confirmatory screening upon identification of carbapenem resistance; (2) CIM as an alternative only where immunochromatographic tests are unavailable; and (3) real-time PCR for cases requiring precise genotypic characterization or when clinical and laboratory findings are discordant. Particularly in settings where OXA-48 and NDM are prevalent, approaches based solely on phenotypic tests are insufficient and should be supplemented by rapid immunochromatographic methods. Multicenter studies involving larger and more heterogeneous sample populations and a broader range of Enterobacterales species are needed to validate and standardize this proposed algorithm.

## Figures and Tables

**Table 1:** Carbapenemase gene profiles of Enterobacterales isolates according to bacterial species.

Gene Profile	*K. pneumoniae* (n = 80)	*E. coli* (n = 16)	Total, n (%)
**Single gene**			
OXA-48	10	7	17 (17.7)
KPC	7	0	7 (7.3)
NDM	5	0	5 (5.2)
**Multiple genes**			
OXA-48 + NDM	53	9	62 (64.6)
OXA-48 + KPC	4	0	4 (4.2)
OXA-48 + NDM + KPC	1	0	1 (1.0)

Abbreviations: KPC, *Klebsiella pneumoniae* carbapenemase; NDM, New Delhi metallo-β-lactamase; OXA-48, oxacillinase-48. Co-production refers to the simultaneous presence of more than one carbapenemase gene within the same isolate.

**Table 2:** Diagnostic performance of the RESIST-5 O.K.N.V.I. test according to carbapenemase type.

Carbapenemase	Sensitivity	Specificity	PPV	NPV	Accuracy
OXA-48	100 (95.7–100)	100 (73.5–100)	100 (95.7–100)	100 (73.5–100)	100 (96.2–100)
NDM	100 (94.5–100)	100 (88.8–100)	100 (94.5–100)	100 (88.8–100)	100 (96.2–100)
KPC	91.7 (61.5–99.8)	100 (95.7–100)	100 (71.5–100)	98.8 (93.5–100)	99.0 (94.3–100)
VIM	N/A	N/A	N/A	N/A	N/A
IMP	N/A	N/A	N/A	N/A	N/A

Confidence intervals (CIs) were calculated using the Clopper–Pearson exact method. Sensitivity, specificity, positive predictive value (PPV), negative predictive value (NPV), and accuracy are presented as percentages (%), with 95% CI shown in parentheses. N/A indicates that calculations could not be performed due to the insufficient number of isolates for the corresponding carbapenemase type.

## Data Availability

The data are not publicly available due to privacy and ethical restrictions but are available from the corresponding author upon reasonable request.

## Supplementary Materials

The following supporting information can be downloaded at: https://www.mdpi.com/article/10.3390/ijms27125454/s1.

## Abbreviations

The following abbreviations are used in this manuscript:

CI	Confidence Interval
CLSI	Clinical and Laboratory Standards Institute
CPE	Carbapenemase-Producing Enterobacterales
EUCAST	European Committee on Antimicrobial Susceptibility Testing
IMP	Imipenemase
KPC	Klebsiella pneumoniae Carbapenemase
MALDI-TOF MS	Matrix-Assisted Laser Desorption/Ionization Time-of-Flight Mass Spectrometry
mCIM	Modified Carbapenem Inactivation Method
NDM	New Delhi Metallo-β-lactamase
NPV	Negative Predictive Value
NPA	Negative Percent Agreement
OPA	Overall Percent Agreement
OXA-48	Oxacillinase-48
PCR	Polymerase Chain Reaction
PPA	Positive Percent Agreement
PPV	Positive Predictive Value
VIM	Verona Integron-Encoded Metallo-β-lactamase
