# Supplementary material for: Comparative Evaluation of Real-Time PCR, Immunochromatographic Assay, and Modified Carbapenem Inactivation Method for Carbapenemase Detection in Enterobacterales Isolates
Source: Int J Mol Sci. 2026 Jun 17;27(12):5454. doi: 10.3390/ijms27125454 (PMC13299616; doi:10.3390/ijms27125454)
Supplement: Supplementary file 1 [file ijms-27-05454-s001.zip › Table S1.pdf]

Table S1. Detailed carbapenemase gene profiles and diagnostic test results of 96 Enterobacterales isolates

Isolate no	Reference (BD MAX™ CPO)	RESIST-5	CIM	Species	Sample Type
1	OXA-48 + NDM	OXA-48 + NDM	Positive	*K. pneumoniae*	Blood
2	KPC	KPC	Positive	*K. pneumoniae*	Blood
3	OXA-48 + NDM	OXA-48 + NDM	Positive	*K. pneumoniae*	Blood
4	OXA-48 + NDM	OXA-48 + NDM	Positive	*K. pneumoniae*	Blood
5	OXA-48 + NDM	OXA-48 + NDM	Positive	*K. pneumoniae*	Blood
6	OXA-48 + NDM	OXA-48 + NDM	Positive	*K. pneumoniae*	Blood
7	OXA-48 + NDM	OXA-48 + NDM	Positive	*K. pneumoniae*	Blood
8	OXA-48 + KPC	OXA-48 + KPC	Positive	*K. pneumoniae*	Blood
9	OXA-48 + NDM	OXA-48 + NDM	Positive	*K. pneumoniae*	Blood
10	OXA-48	OXA-48	Positive	*E. coli*	Blood
11	OXA-48 + NDM + KPC	OXA-48 + NDM	Positive	*K. pneumoniae*	Blood
12	OXA-48	OXA-48	Negative	*E. coli*	Blood
13	OXA-48 + NDM	OXA-48 + NDM	Positive	*K. pneumoniae*	Blood
14	KPC	KPC	Positive	*K. pneumoniae*	Blood
15	OXA-48 + NDM	OXA-48 + NDM	Positive	*E. coli*	Blood
16	OXA-48	OXA-48	Positive	*K. pneumoniae*	Blood
17	OXA-48 + NDM	OXA-48 + NDM	Positive	*K. pneumoniae*	Blood
18	NDM	NDM	Positive	*K. pneumoniae*	Blood
19	OXA-48 + NDM	OXA-48 + NDM	Positive	*K. pneumoniae*	Blood
20	OXA-48 + NDM	OXA-48 + NDM	Positive	*K. pneumoniae*	Blood
21	OXA-48 + KPC	OXA-48 + KPC	Positive	*K. pneumoniae*	Blood
22	OXA-48	OXA-48	Positive	*E. coli*	Urine
23	OXA-48 + NDM	OXA-48 + NDM	Positive	*K. pneumoniae*	Urine
24	OXA-48 + NDM	OXA-48 + NDM	Positive	*K. pneumoniae*	Urine
25	OXA-48	OXA-48	Positive	*E. coli*	Urine
26	OXA-48 + NDM	OXA-48 + NDM	Positive	*K. pneumoniae*	Urine
27	OXA-48 + NDM	OXA-48 + NDM	Positive	*E. coli*	Urine
28	OXA-48	OXA-48	Positive	*K. pneumoniae*	Urine
29	OXA-48 + NDM	OXA-48 + NDM	Positive	*K. pneumoniae*	Blood
30	NDM	NDM	Negative	*K. pneumoniae*	Blood
31	OXA-48	OXA-48	Positive	*K. pneumoniae*	Blood
32	OXA-48 + NDM	OXA-48 + NDM	Positive	*E. coli*	Blood
33	KPC	KPC	Positive	*K. pneumoniae*	Blood
34	OXA-48 + NDM	OXA-48 + NDM	Positive	*E. coli*	Tracheal aspirate
35	OXA-48 + NDM	OXA-48 + NDM	Positive	*K. pneumoniae*	Tracheal aspirate
36	OXA-48 + NDM	OXA-48 + NDM	Positive	*K. pneumoniae*	Tracheal aspirate
37	OXA-48 + NDM	OXA-48 + NDM	Positive	*K. pneumoniae*	Tracheal aspirate
38	OXA-48 + NDM	OXA-48 + NDM	Positive	*K. pneumoniae*	Tracheal aspirate
39	OXA-48 + NDM	OXA-48 + NDM	Positive	*K. pneumoniae*	Tracheal aspirate
40	OXA-48 + NDM	OXA-48 + NDM	Positive	*K. pneumoniae*	Tracheal aspirate
41	OXA-48 + NDM	OXA-48 + NDM	Positive	*K. pneumoniae*	Tracheal aspirate
42	KPC	KPC	Positive	*K. pneumoniae*	Tracheal aspirate
43	OXA-48 + NDM	OXA-48 + NDM	Positive	*E. coli*	Tracheal aspirate
44	OXA-48 + NDM	OXA-48 + NDM	Positive	*K. pneumoniae*	Tracheal aspirate
45	OXA-48 + NDM	OXA-48 + NDM	Positive	*K. pneumoniae*	Tracheal aspirate
46	OXA-48 + NDM	OXA-48 + NDM	Positive	*K. pneumoniae*	Tracheal aspirate
47	OXA-48 + NDM	OXA-48 + NDM	Positive	*K. pneumoniae*	Tracheal aspirate
48	OXA-48 + KPC	OXA-48 + KPC	Positive	*K. pneumoniae*	Sputum

49	OXA-48 + NDM	OXA-48 + NDM	Positive	*E. coli*	Sputum
50	OXA-48	OXA-48	Positive	*E. coli*	Sputum
51	OXA-48 + NDM	OXA-48 + NDM	Positive	*K. pneumoniae*	Sputum
52	OXA-48	OXA-48	Positive	*E. coli*	Wound
53	OXA-48 + NDM	OXA-48 + NDM	Positive	*K. pneumoniae*	Wound
54	KPC	KPC	Positive	*K. pneumoniae*	Wound
55	OXA-48 + NDM	OXA-48 + NDM	Positive	*K. pneumoniae*	Wound
56	OXA-48	OXA-48	Positive	*K. pneumoniae*	Wound
57	OXA-48 + NDM	OXA-48 + NDM	Positive	*K. pneumoniae*	Blood
58	NDM	NDM	Positive	*K. pneumoniae*	Blood
59	OXA-48 + NDM	OXA-48 + NDM	Positive	*K. pneumoniae*	Blood
60	OXA-48 + NDM	OXA-48 + NDM	Positive	*K. pneumoniae*	Blood
61	OXA-48 + KPC	OXA-48 + KPC	Positive	*K. pneumoniae*	Blood
62	OXA-48	OXA-48	Positive	*E. coli*	Blood
63	OXA-48 + NDM	OXA-48 + NDM	Positive	*K. pneumoniae*	Blood
64	OXA-48 + NDM	OXA-48 + NDM	Positive	*K. pneumoniae*	Blood
65	OXA-48	OXA-48	Positive	*K. pneumoniae*	Blood
66	OXA-48 + NDM	OXA-48 + NDM	Positive	*E. coli*	Blood
67	OXA-48 + NDM	OXA-48 + NDM	Positive	*K. pneumoniae*	Blood
68	OXA-48	OXA-48	Positive	*K. pneumoniae*	Blood
69	OXA-48 + NDM	OXA-48 + NDM	Positive	*K. pneumoniae*	Blood
70	NDM	NDM	Negative	*K. pneumoniae*	Blood
71	OXA-48	OXA-48	Positive	*K. pneumoniae*	Blood
72	KPC	KPC	Positive	*K. pneumoniae*	Blood
73	OXA-48 + NDM	OXA-48 + NDM	Positive	*K. pneumoniae*	Tracheal aspirate
74	OXA-48 + NDM	OXA-48 + NDM	Positive	*K. pneumoniae*	Tracheal aspirate
75	OXA-48 + NDM	OXA-48 + NDM	Positive	*K. pneumoniae*	Tracheal aspirate
76	OXA-48 + NDM	OXA-48 + NDM	Positive	*E. coli*	Blood
77	OXA-48 + NDM	OXA-48 + NDM	Positive	*E. coli*	Blood
78	OXA-48 + NDM	OXA-48 + NDM	Positive	*K. pneumoniae*	Blood
79	OXA-48 + NDM	OXA-48 + NDM	Positive	*K. pneumoniae*	Blood
80	OXA-48 + NDM	OXA-48 + NDM	Positive	*K. pneumoniae*	Blood
81	OXA-48 + NDM	OXA-48 + NDM	Positive	*K. pneumoniae*	Blood
82	OXA-48	OXA-48	Positive	*K. pneumoniae*	Blood
83	OXA-48 + NDM	OXA-48 + NDM	Positive	*K. pneumoniae*	Blood
84	OXA-48 + NDM	OXA-48 + NDM	Positive	*K. pneumoniae*	Blood
85	OXA-48	OXA-48	Positive	*K. pneumoniae*	Blood
86	OXA-48 + NDM	OXA-48 + NDM	Positive	*K. pneumoniae*	Blood
87	NDM	NDM	Positive	*K. pneumoniae*	Blood
88	OXA-48	OXA-48	Positive	*K. pneumoniae*	Blood
89	KPC	KPC	Positive	*K. pneumoniae*	Blood
90	OXA-48 + NDM	OXA-48 + NDM	Positive	*K. pneumoniae*	Blood
91	OXA-48 + NDM	OXA-48 + NDM	Positive	*K. pneumoniae*	Blood
92	OXA-48 + NDM	OXA-48 + NDM	Positive	*K. pneumoniae*	Blood
93	OXA-48 + NDM	OXA-48 + NDM	Positive	*K. pneumoniae*	Urine
94	OXA-48 + NDM	OXA-48 + NDM	Positive	*K. pneumoniae*	Urine
95	OXA-48 + NDM	OXA-48 + NDM	Positive	*K. pneumoniae*	Urine
96	OXA-48 + NDM	OXA-48 + NDM	Positive	*K. pneumoniae*	Urine

Isolate 11: KPC was not detected by the RESIST-5 test. Isolates 12 and 30: the CIM test yielded false-negative results.
